# How physical exercise enhances life satisfaction in Chinese senior college students: mediating roles of self-efficacy and resilience

**DOI:** 10.3389/fpsyg.2025.1515101

**Published:** 2025-02-21

**Authors:** Huasen Yu, Xuening Li, Xin Yu, Liang Fusheng, Liqiang Li, Yuhang Yang, Jin Wu

**Affiliations:** ^1^School of Physical Education and Health, East China Normal University, Shanghai, China; ^2^Department of Physical Education, Nanjing University of Posts and Telecommunications, Nanjing, China; ^3^School of Physical Education, Nanchang Normal University, Nanchang, Jiangxi, China; ^4^Department of Physical Education, Huangshan University, Huangshan, China; ^5^School of Physical Education, Xizang Minzu University, Xianyang, China; ^6^Department of Physical Education, College of Education, Zhejiang University, Hangzhou, China

**Keywords:** senior college students, physical exercise, life satisfaction, self-efficacy, psychological resilience

## Abstract

**Objective:**

This study explored the relationship between physical exercise and life satisfaction among senior college students by focusing on the mediating roles of self-efficacy and resilience, thereby providing theoretical support for the mental health benefits of physical exercise for senior students.

**Methods:**

A survey was conducted with 600 senior students from 10 universities using the physical exercise rating, life satisfaction, self-efficacy, and resilience scales. Data analysis was performed using SPSS 23.0 for descriptive statistics, correlation analysis, and reliability and validity assessments. In addition, AMOS software (version 24.0) was used to construct a structural equation model to test the hypothesized pathways and the effects of potential mediating variables.

**Results:**

There were significant positive correlations among the variables of physical exercise, life satisfaction, self-efficacy, and resilience (r = 0.11–0.62, all *p* < 0.01). The structural equation model revealed that resilience fully mediated the relationship between physical exercise and life satisfaction among senior students (95% CI: 0.105–0.243), while self-efficacy did not mediate this relationship (β = 0.02, *p* = 0.77). However, a chain mediating effect involving self-efficacy and resilience was observed between physical exercise and life satisfaction (95% CI: 0.127–0.235).

**Conclusion:**

Enhanced physical exercise can improve self-efficacy and resilience among senior college students, thereby increasing their life satisfaction.

## 1 Introduction

There is a significant increase in the prevalence of depressive symptoms among Chinese college students, rising from 23.8% before 2019 to 26.0% in 2021 (Luo et al., 2021). Concurrently, anxiety symptoms affect nearly a quarter of college students, driven by economic downturns and declining employment rates (Wang and Liu, 2022). Senior college students, on the verge of transitioning from academia to professional life, face unique pressures such as completing theses, adapting to lifestyle changes, and navigating a competitive job market. Such stressors markedly diminish their life satisfaction and heighten psychological distress (Shan et al., 2022). This highlights an urgent need for research and interventions tailored to address these specific vulnerabilities.

### 1.1 Physical exercise and life satisfaction

Physical exercise positively influences life satisfaction through enhancements in psychological wellbeing (Kekäläinen et al., 2020; Herbert, 2022), emotional states (Ram et al., 2022; Waaso et al., 2022), and social engagement (Zhang et al., 2023). Meyer et al. (2021) emphasize that regular physical activity not only reduces anxiety but also fosters emotional regulation, particularly among high-stress populations like college students (Meyer et al., 2021). These findings underscore the importance of regular physical activity to foster improved mental health and overall life satisfaction. A large-scale analysis based on data, comprising 51,977 participants, similarly found that the relationship between physical exercise and life satisfaction is not always direct (Zhong and Xu, 2024). As Nam et al. found that self-determined motivation could be a key factor in exercise adherence and mental health outcomes, emphasizing the influence of contextual factors such as exercise environments and individual differences (Nam et al., 2023). Moreover, cultural, individual, and socio-economic factors have been found to moderate this relationship, leading to variability in findings across different populations (Brailovskaia et al., 2022). These findings not only highlight the multifactorial nature of the impact of physical exercise on life satisfaction but also underscore the need for further exploration of its underlying mechanisms and mediating variables.

### 1.2 Relationship between physical exercise, self-efficacy, and life satisfaction

Self-efficacy, defined as an individual's belief in their ability to execute actions to achieve desired outcomes (Bandura, 1993), plays a pivotal role in stress management and psychological wellbeing. Regular engagement in physical exercise enhances an individual's capacity to attain preset goals and reinforces self-affirmation of capabilities, thereby elevating self-efficacy (Tikac et al., 2022). Evidence further suggests that exercise behavior acts as an important mediator in the relationship between physical activity and self-efficacy, highlighting the value of improving exercise behaviors among college students (Han et al., 2022). Empirical studies by domestic and international scholars have shown that self-efficacy plays a significant role in the development of life satisfaction among college students (Mao et al., 2023). Higher self-efficacy is associated with increased life satisfaction in college students, with a direct positive relationship between the two (Wilcox and Nordstokke, 2019), making self-efficacy an important predictor of life satisfaction (Wang et al., 2022a,b). High self-efficacy promotes adaptive coping strategies and fosters a sense of control, thereby enhancing life satisfaction (Luo, 2024). Overall, existing evidence suggests that self-efficacy may play a crucial mediating role in the relationship between physical exercise and life satisfaction.

### 1.3 Relationship between physical exercise, resilience, and life satisfaction

Resilience, known as “psychological resilience” or “psychological hardiness” (Elavsky and McAuley, 2005), characterized by the capacity to adapt positively to adversity, acts as a psychological buffer that mitigates the adverse effects of stress and promotes emotional stability (Connor and Davidson, 2003). On the one hand, research has found that physical exercise plays a crucial role in building resilience (Xu et al., 2021). Li et al. (2024) revealed that individuals who engage in regular physical exercise are more likely to develop high levels of resilience. According to Belcher et al. (2021), physical exercise enhances resilience through mechanisms involving brain development and self-regulation. On the another hand, numerous studies have confirmed that resilience is significantly and positively correlated with life satisfaction (Shi et al., 2015; Wang et al., 2022b). Resilience improves life satisfaction by fulfilling basic psychological needs, such as autonomy, competence, and relatedness (Xu et al., 2021). Longitudinal studies further confirmed the unidirectional predictive relationship between resilience and life satisfaction, showing that high levels of resilience effectively buffer stress and reduce academic burnout, thereby enhancing life satisfaction (Wang et al., 2022a). Overall, this evidence provided a better understanding and valuable insights into aspects of resilience as potential mediators of in the relationship between physical.

### 1.4 Relationship between physical exercise, self-efficacy, resilience and life satisfaction

Interestingly, some literature highlights the association between resilience and self-efficacy among students (Sabouripour et al., 2021). Self-efficacy has been identified as a significant predictor of resilience, as higher self-efficacy enhances individuals' capacity to cope with stress and adapt positively to challenges (Xu et al., 2021; Li et al., 2024). This relationship underscores the importance of self-efficacy in fostering resilience, suggesting a sequential link between these constructs. Self-efficacy and resilience may serve as mediating factors in the relationship between physical exercise and life satisfaction. Physical exercise enhances self-efficacy by reinforcing individuals' belief in their capabilities, which, in turn, strengthens resilience (Li et al., 2024). Resilience further supports emotional stability and stress management, contributing to higher life satisfaction. This serial mediation pathway not only explains the psychological mechanisms underlying the effects of physical exercise but also highlights the interconnected roles of self-efficacy and resilience in promoting wellbeing (Belcher et al., 2021; Zhang et al., 2022).

### 1.5 The present study

While prior studies have established the mediating roles of self-efficacy and resilience, they have predominantly focused on general populations or specific groups such as younger students or elite athletes (Morales-Rodríguez et al., 2020; Sabouripour et al., 2021). Research addressing these mediating mechanisms in the context of senior college students remains scarce, despite their unique challenges and heightened vulnerability during this transitional period. This study integrates psychological constructs and behavioral factors to provide a comprehensive framework for understanding how physical exercise supports life satisfaction for senior college students during this critical transitional phase.

Building on these insights, the present study aims to address two key objectives: (1) examining the relationships among physical exercise, life satisfaction, self-efficacy, and resilience, and (2) investigating the chain-mediating roles of self-efficacy and resilience in the relationship between physical exercise and life satisfaction. The findings are expected to inform targeted interventions, such as resilience training and adaptive coping strategies, to enhance the wellbeing of this vulnerable population.

## 2 Methods

### 2.1 Participants and procedure

#### 2.1.1 Participant recruitment

Participants were recruited for this study from June 1 to 18, 2024 via a WeChat messaging application. The study utilized a simple random sampling method, aiming to select an easily accessible and representative sample. Given the vast geographical expanse of China, along with regional variations in economic, cultural, and educational backgrounds, four representative provinces were chosen: Shanghai, Henan, Jilin, and Liaoning. In each province, 2–3 universities were randomly selected from each of the two types of comprehensive universities and local colleges. In addition, senior students were selected as the target group, who are at the critical stage of academia to the workforce. Their life satisfaction and psychological status may be significantly affected by multiple factors such as academic pressure and employment anxiety. To mitigate potential confounding factors, students who had already been admitted to graduate school were excluded from the study, as their life satisfaction and psychological status may differ markedly from those of students facing graduation and the imminent challenges of entering the job market.

#### 2.1.2 Questionnaire distribution and completion

The survey was conducted using the “Wen juanxing” platform, which created an online questionnaire that was distributed to participants via WeChat. All participants completed the questionnaire on their mobile devices and provided electronic informed consent prior to participation. The consent form outlined the voluntary nature of participation, guaranteed the confidentiality of data, and confirmed that participation would not affect academic performance. The design and content of the questionnaire were grounded in relevant literature, ensuring methodological rigor reproducibility.

#### 2.1.3 Data quality and statistical assessment

A total of 643 questionnaires were distributed, and after excluding 43 invalid questionnaires, 600 valid questionnaires were finally returned, yielding a valid return rate of 93.31%. Invalid questionnaires were identified based on criteria such as unusually short response time (< 2 min), patterns of repetitive answers, or missing key questions. To ensure data quality, the research team enlisted a professional statistician to oversee the distribution, collection, and data cleaning process, thereby ensuring the scientific rigor and reliability of the findings.

#### 2.1.4 Participant characteristics

The mean age of the participants who completed the valid questionnaire was 22.63 ± 0.73 years. Among them, 158 (26.33%) were majoring in science and engineering, while 442 (73.67%) were majoring in liberal arts. In terms of gender, 235 (39.17%) were male, and 365 (60.83%) were female. The questionnaire included physical activity, life satisfaction, self-efficacy, and resilience scales. All of these scales well-established tools in psychological research and have demonstrated good reliability and validity. The questionnaire took about 5–10 min to complete, and the research process was designed to ensure a positive participant experience and efficient data collection.

The study was conducted in a accordance with the ethical standards set force in the 1964 Declaration of Helsinki and its subsequent revisions, and was approved by the Ethics Committee of East China Normal University (HR284-2024).

### 2.2 Measurement tools

#### 2.2.1 Physical activity rating scale

The physical activity rating scale, originally developed by Japanese psychologist Hashimoto Kio and later revised by Liang Deqing, was utilized to assess participants' physical activity levels (Liang, 1994). This scale comprises three components that measure the intensity, frequency, and duration of physical exercise. The specific calculation formula was as follows: physical activity level = intensity × duration × frequency. The intensity and frequency levels ranged from 1 (1 point) to 5 (5 points), and the duration ranged from 1 (0 points) to 5 (4 points). Total scores on the physical activity scale range from 0 to 100. According to scoring standards, physical activity levels were categorized as low ( ≤ 19 points), moderate (20–42 points), and high activities (≥43 points). This scale has good reliability with a total coefficient of 0.82 (Liang, 1994). In this study, the test-retest reliability of the physical activity rating scale was 0.820.

#### 2.2.2 Satisfaction with life scale

The satisfaction with life scale developed by Diener et al. (1985) was used to assess participants' life satisfaction. This scale includes 5 items, each scored on a 7-point scale ranging from 1 (very dissatisfied) to 7 (very satisfied). Higher total scores indicate higher life satisfaction. The scale has shown good reliability and validity in tests with college students, with a Cronbach's alpha of 0.885. Confirmatory factor analysis supported the structure of this questionnaire, χ^2^ = 10.71, df = 4, χ^2^/df = 2.68, GFI = 0.99, NFI = 0.97, CFI = 0.99, RMSEA = 0.05, with factor loadings for each item ranging from 0.45 to 0.90, indicating good structural validity. In this study, Cronbach's alpha was 0.844.

#### 2.2.3 General self-efficacy scale

This study used the general self-efficacy scale developed by Schwarzer et al. and revised into Chinese by Caikang et al. to measure participants' self-efficacy (Schwarzer et al., 1999). The scale includes 10 items, each scored on a 4-point scale ranging from 1 (not at all true) to 4 (exactly true). Higher total scores indicate higher general self-efficacy. This scale is widely used in research on college students, with a Cronbach's alpha of 0.87 (Wang et al., 2001). Confirmatory factor analysis supported the structure of this questionnaire, χ^2^ = 197.32, df = 35, χ^2^/df = 5.63, GFI = 0.94, NFI=0.92, CFI=0.93, RMSEA=0.08, with factor loadings for each item ranging from 0.47 to 0.76. In this study, Cronbach's alpha was 0.883.

#### 2.2.4 Connor-Davidson resilience scale

The Connor-Davidson resilience scale, developed by Connor and Davidson in 2003 and revised into Chinese by Nan and Jianxin, was used to measure participants' resilience. This scale includes 25 items that cover three dimensions: tenacity, strength, and optimism. Each item is scored on a 5-point scale ranging from 1 (not at all true) to 5 (true nearly all the time). Higher total scores indicated higher levels of resilience. The scale had a Cronbach's alpha of 0.915 (Yu and Zhang, 2007). Owing to the item “I have to act on a hunch” in the tenacity dimension with a factor loading of 0.23, which is below the minimum standard of 0.40, this item (S10) was removed. Confirmatory factor analysis supported the structure of the questionnaire, with major fit indices χ^2^ = 469.27, df = 227, χ^2^/df = 2.07, GFI = 0.94, NFI = 0.91, CFI = 0.95, RMSEA = 0.06. In this study, Cronbach's alpha coefficients for tenacity, strength, and optimism were 0.873, 0.803, and 0.616, respectively.

### 2.3 Statistical methods

In this study, SPSS 23.0 software was used for statistical data analysis, and AMOS software (version 24.0) was used to test the mediation model, employing the bias-corrected percentile bootstrap method for mediating effect testing. The data analysis process involves several steps. First, data pre-processing was performed using SPSS 23.0, including data importation and descriptive statistical analyses. In addition, a correlation analysis among the variables was conducted using Pearson's correlation coefficients to calculate the linear relationships between variables, generating a correlation matrix to identify significantly correlated variables. Second, AMOS software (version 24.0) was used to construct a structural equation model (SEM) to explore the mechanisms through which physical exercise influences life satisfaction. During the model fitting process, the fit indices of the model (such as χ^2^/df, GFI, AGFI, CFI, NFI, and RMSEA) were examined. The model structure was adjusted based on the fit results to improve rationality (Bentler, 1990; Lin et al., 2021). The bias-corrected percentile bootstrap method was used to test for the mediating effect. The number of bootstrap samples was set to 5,000. The distribution of each path coefficient was calculated using bootstrap sampling, and 95% confidence intervals for the path coefficients were computed. The bias-corrected percentile method was used to adjust confidence intervals and ensure accuracy of the estimates. A confidence interval that did not contain zero indicated a significant mediating effect (Li et al., 2024). By reporting the size and significance of the mediating effects, the mechanisms by which the independent variables influenced the dependent variables through the mediator variables were explained, thereby providing empirical support for our research hypotheses.

## 3 Results

### 3.1 Control and examination of common method bias

To address the potential risk of common method bias (CMB) arising from self-reported data collection, this study implemented a combination of procedural and statistical controls. Procedurally, the questionnaire was designed to minimize bias through psychological and methodological separation (Kock et al., 2021). Independent and dependent variable items were placed in distinct sections to reduce overlap, and reverse-coded items alongside mixed Likert scales (e.g., 5-point, and 7-point scales) were incorporated to mitigate response pattern bias (Podsakoff et al., 2024). Anonymity of responses was ensured with explicit instructions to reduce social desirability bias.

Harman's single-factor test was performed as a preliminary assessment, revealing that the first factor accounted for 27.72% of the total variance, below the 40% threshold. To further ensure robustness, the unmeasured latent method construct technique was applied within a structural equation modeling (SEM) framework. By adding a latent method factor to account for shared variance among indicators, results confirmed that CMB had minimal impact on the study's findings. These combined approaches reinforced the validity and reliability of the conclusions.

### 3.2 Descriptive statistics and correlation analysis

Correlation analysis was conducted for physical exercise, life satisfaction, self-efficacy, and resilience (Table 1). The results revealed significant positive correlations between physical exercise and life satisfaction (r = 0.15, *p* < 0.01), self-efficacy (r = 0.11, *p* < 0.01), and resilience (r = 0.25, *p* < 0.01). These findings indicated that higher levels of physical exercise are associated with higher levers of life satisfaction, self-efficacy, and resilience. Life satisfaction also positively correlated with self-efficacy (r = 0.33, *p* < 0.01) and resilience (r = 0.44, *p* < 0.01), suggesting their interconnected influence. Furthermore, self-efficacy was strongly correlated with resilience (r = 0.62, *p* < 0.01), highlighting its potential role in resilience development. However, no significant relationships were found between exercise duration and either life satisfaction (r = 0.06, *p* > 0.05) or self-efficacy (r = 0.05, *p* > 0.05), nor between exercise frequency and self-efficacy (r = 0.02, *p* > 0.05). These results suggested that while physical exercise as a whole positively impacts psychological outcomes, specific exercise dimensions may vary in their effects. Further studies are needed to examine these nuanced relationships.

**Table 1:** Descriptive statistics and correlation analysis of main variables.

	**M ±SD**	**1**	**2**	**3**	**4**	**5**	**6**	**7**
1. Total physical exercise	23.8 ± 23.74	1.00						
2. Exercise intensity	2.43 ± 1.30	0.78^**^	1.00					
3. Exercise time	2.50 ± 1.22	0.69^**^	0.41^**^	1.00				
4. Exercise frequency	3.25 ± 1.12	0.54^**^	0.22^**^	0.32^**^	1.00			
5. Life satisfaction	18.40 ± 6.29	0.15^**^	0.17^**^	0.06	0.09	1.00		
6. Self-efficacy	26.35 ± 5.18	0.11^**^	0.16^**^	0.05	0.02	0.33^**^	1.00	
7. Resilience	83.2 ± 13.08	0.25^**^	0.20^**^	0.17^**^	0.19^**^	0.44^**^	0.62^**^	1.00

^**^p < 0.01.

### 3.3 Impact of different dimensions of physical exercise on life satisfaction

These findings suggest that physical exercise did not have a direct positive influence on life satisfaction. However, the specific impacts of these dimensions on life satisfaction remain unclear. Stepwise regression was used to further clarify this impact. In this method, each introduced independent variable is tested against the variables already in the equation, and the variables that meet the exclusion criteria are removed individually. The ΔR^2^ value of each predictor variable is calculated through hierarchical stepwise regression to examine the relative contribution of each dimension of physical exercise to the total variance. The results indicated that exercise intensity significantly predicted life satisfaction, with R^2^ = 0.029 (adjusted R^2^ = 0.027, ΔR^2^ = 0.029, *p* = 0.000). This suggests that exercise intensity accounts for 2.9% of the variance in life satisfaction among senior college students. However, the dimensions of exercise duration and frequency were not significantly correlated with life satisfaction (*p* > 0.05) and were thus excluded from the model. These findings demonstrate that among the examined dimensions, exercise intensity plays a unique yet limited role in predicting life satisfaction. The exclusion of exercise duration and frequency underscores the complexity of physical exercise's effects on psychological outcomes, suggesting that other unexamined factors may mediate or moderate these relationships. This aligns with previous research indicating that not all forms or aspects of physical activity uniformly influence wellbeing outcomes. Future studies should explore these dimensions in greater depth to better understand their nuanced impacts.

### 3.4 Testing the hypothetical model of the mediating role of self-efficacy and resilience

To analyze the relationships among variables, structural equation modeling (SEM) was applied using AMOS 24.0. Considering that life satisfaction and self-efficacy were measured using unidimensional scales comprising numerous items, the latent variables were aggregated before constructing the model to mitigate inflated measurement errors caused by multiple items (Ledgerwood and Shrout, 2011). The variable aggregation followed the factor method, ensuring unidimensionality and homogeneity of the scales (Matsunaga, 2008). After aggregation, self-efficacy was packed into three parcels: Efficacy 1, Efficacy 2, and Efficacy 3, while life satisfaction was packed into two parcels: Satisfaction 1 and Satisfaction 2.

**Figure 1:**
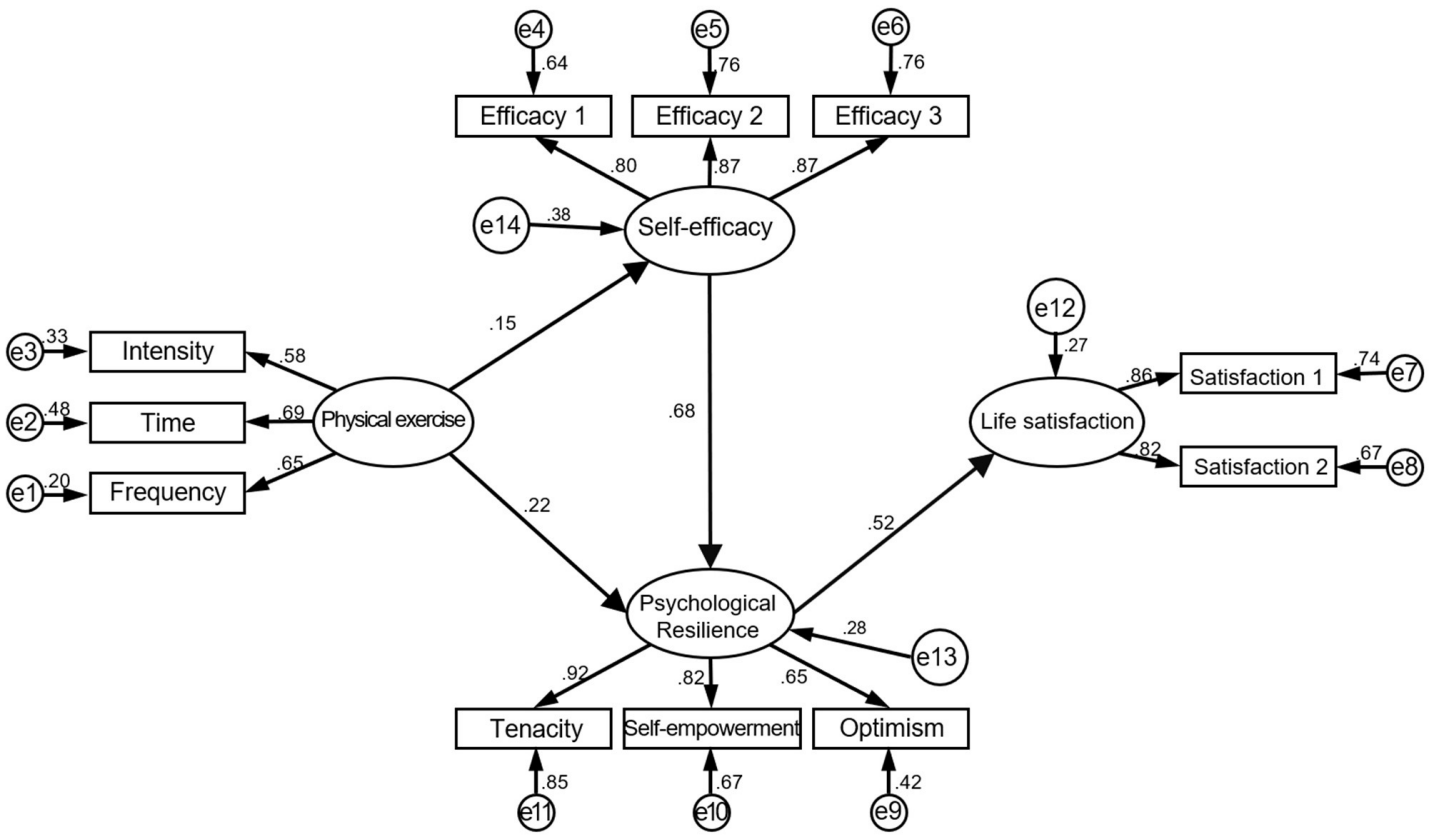
Initial model of the relationships between physical exercise, self-efficacy, resilience, and life satisfaction.

**Figure 2:**
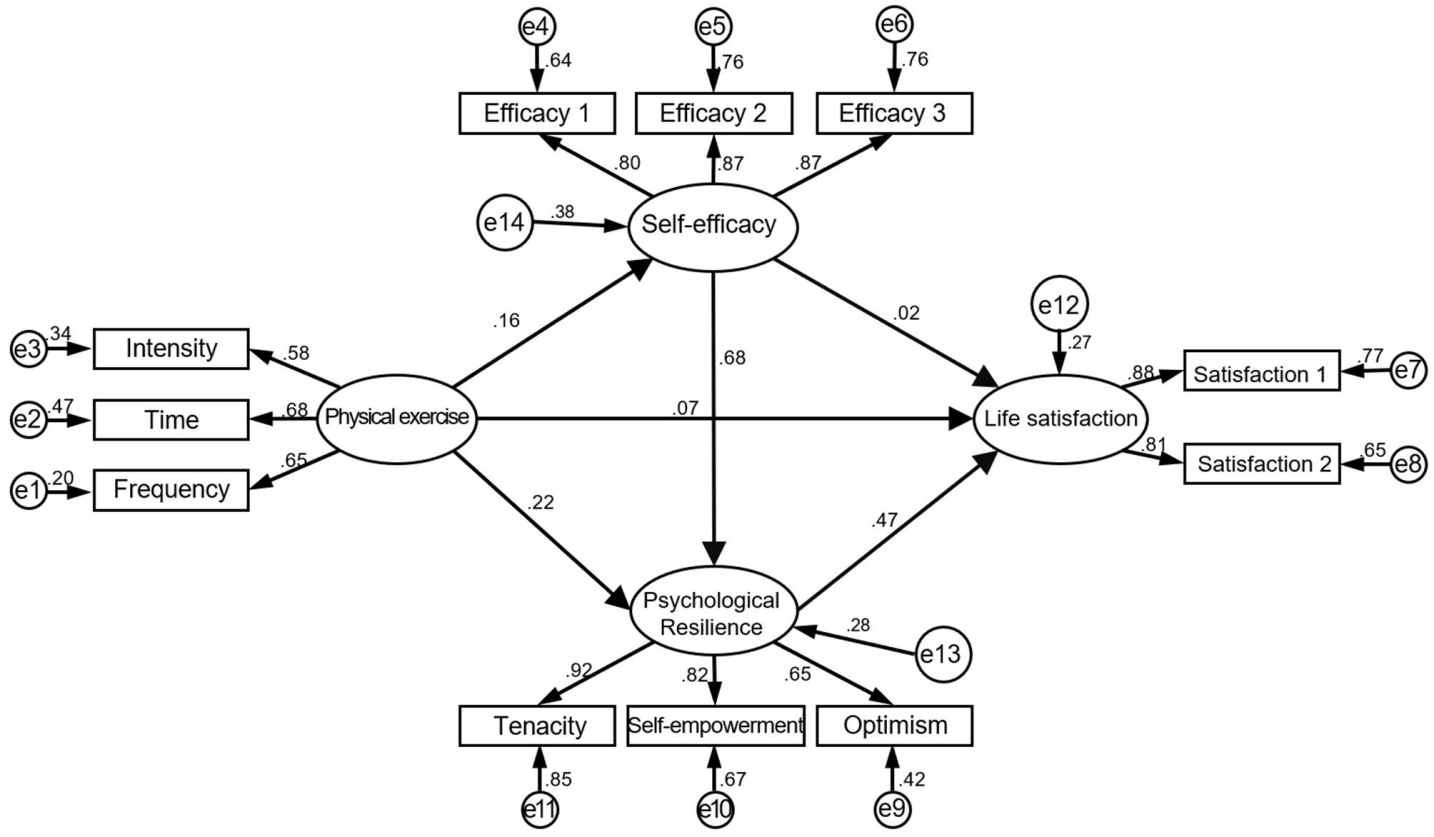
Modified model of the relationships between physical exercise, self-efficacy, resilience, and life satisfaction.

The initial SEM model (Figure 1) was constructed based on proposed hypotheses and variable correlations. The model's fit indices were χ^2^/df = 4.62, GFI = 0.95, AGFI = 0.91, CFI = 0.95, NFI = 0.94, and RMSEA = 0.08. However, further analysis revealed that the direct path between physical exercise and life satisfaction (β = 0.07, t = 1.26, *P* = 0.21) and the path from self-efficacy to life satisfaction (β = 0.02, t = 0.29, *P* = 0.77) were not significant. After removing these paths, a revised model (Figure 2) was obtained. The revised model showed improved fit indices: χ^2^/df = 4.42, GFI = 0.95, AGFI = 0.91, CFI = 0.96, NFI = 0.94, and RMSEA = 0.08. The revised model indicated better alignment with the observed data, supporting the hypothesis that resilience plays a key mediating role between physical exercise and life satisfaction.

### 3.5 Mediating effect testing

This study used the bootstrap method to test the mediating effects in the structural model. The bias-corrected percentile bootstrap method was employed with 5,000 resamples, and the confidence interval for the mediating effect was set at 95%. As shown in Table 2, the total effect of physical exercise on life satisfaction was significant (β = 0.16, *p* < 0.05), and the bias-corrected confidence interval did not include zero. In addition, the 95% confidence intervals for the impact paths in the revised model (Figure 2) excluded zero, indicating significant mediating effects. Specifically, the mediating effect of resilience between physical exercise and life satisfaction was significant (β = 0.11, *p* < 0.05). Similarly, the mediating effect of resilience on self-efficacy and life satisfaction was significant (β = 0.35, *p* < 0.05). Moreover, the chain mediating effect of self-efficacy and resilience on physical exercise and life satisfaction was significant (β = 0.05, *p* < 0.05).

**Table 2:** Bootstrap analysis of mediating effect testing.

**Impact path**	**Standardized effect value**	**95% confidence interval**	
		**Lower limit**	**Upper limit**
Total effect: Physical exercise → Life satisfaction	0.16	0.247	0.632
Mediating effect: Physical exercise → Resilience → Life satisfaction	0.11	0.105	0.243
Physical exercise → Self-efficacy → Resilience	0.10	0.021	0.187
Self-efficacy → Resilience → Life Satisfaction	0.35	0.288	0.424
Chain mediating effect: Physical exercise → Self-efficacy → Resilience → Life satisfaction	0.05	0.127	0.235

## 4 Discussion

### 4.1 Impact of physical exercise on life satisfaction

Correlation analysis showed significant positive correlations among exercise intensity, time, frequency, and life satisfaction, with only exercise intensity exerting a significant impact on life satisfaction. Therefore, the predictive effect of physical exercise on the life satisfaction of senior students is minimal. This finding aligns with prior research (Zhong and Xu, 2024), suggesting that physical exercise does not always directly predict life satisfaction. The low predictive effect of physical exercise on life satisfaction may be due to two factors. On one hand, researchers found that only high-intensity exercise can significantly promote dopamine release, improve emotional states, and enhance subjective wellbeing (Davis et al., 2011). However, the levels of physical exercise among senior students in this study were relatively low, failing to improve their physical and mental states, and thus had a weak impact on life satisfaction. On the other hand, life satisfaction is an individual's subjective evaluation of overall life quality, while physical exercise more directly provides pleasurable and fulfilling experiences both physically and mentally. These positive experiences may indirectly influence life satisfaction by improving an individual's perception of health and happiness. Most importantly, the effects of exercise frequency and duration may be weakened for the senior students due to academic pressure and time constraints. The weak direct relationship between exercise frequency, duration, and life satisfaction in this study indicates that future research should explore the differential effects of various exercise durations, frequencies, and intensities on psychological health.

### 4.2 Mediating role of resilience between physical exercise and life satisfaction

This study showed that resilience plays a significant mediating role between physical exercise and life satisfaction. The analysis revealed that physical exercise significantly enhances resilience, which in turn positively predicts life satisfaction. This result aligns with previous studies, indicating that regular participation in physical activities can enhance an individual's adaptability and resilience (Xu et al., 2021). Resilience, a crucial psychological resource, helps senior students cope better with multiple pressures during the graduation transition. For example, when faced with job-search pressures or academic tasks, resilience can help reduce negative emotions caused by setbacks, enhance problem-solving abilities, and promote positive psychological regulation (Chang et al., 2023). Additionally, the role of resilience in buffering negative emotions (such as anxiety and depression) has been validated (Wang et al., 2022a). This buffering effect not only reduces the individual's negative evaluations of life situations but also enhances their life satisfaction. Given the results of this study, resilience should be recognized as a vital form of health capital. Future intervention measures should combine physical exercise with psychological skills training to help university students enhance resilience and adapt to the multiple challenges of the graduation transition.

### 4.3 Chain mediating role of self-efficacy and resilience in the relationship between physical exercise and life satisfaction

This study further revealed that self-efficacy and resilience have a chain-mediated effect on the relationship between physical exercise and life satisfaction. Specifically, physical exercise enhances self-efficacy, which in turn significantly improves resilience, and ultimately promotes life satisfaction through this chain path. Self-efficacy, a core concept in Bandura's social cognitive theory (Bandura, 1993), indicates that regular participation in physical exercise enhances individuals' confidence in achieving goals and their ability to cope with complex situations (Tikac et al., 2022). High self-efficacy not only promotes the adoption of adaptive coping strategies but also reduces psychological distress, laying the foundation for improving resilience (Sabouripour et al., 2021). For senior students, the confidence gained from physical exercise helps them cope more effectively with the dual pressures of academic work and employment, while also providing valuable psychological resources for solving complex problems and alleviating psychological distress. Resilience further serves as a mediator in the relationship between self-efficacy and life satisfaction. Resilience improves emotional stability and rapid recovery abilities, helping students bounce back from setbacks quickly (Zhang et al., 2023), and providing greater adaptability and coping strategies when facing major life changes, such as leaving campus and entering society. In the current economic context of a downturn and declining employment rates, this psychological support is particularly important, providing a key safeguard for enhancing life satisfaction. This chain-mediated effect reveals the layered accumulation path of psychological capital and demonstrates how physical exercise indirectly influences life satisfaction through self-efficacy and resilience. The results suggest that future interventions should integrate short, efficient physical activities with psychological skills training to help students better cope with the complex challenges of the graduation transition and improve life satisfaction.

## 5 Limitations and future directions

This study systematically explores the psychological mechanisms between physical exercise and life satisfaction from the perspectives of self-efficacy and resilience using structural equation modeling. It contributes to enriching theoretical knowledge and provides important practical guidance for improving the mental health and life satisfaction of senior university students. However, this study has several limitations:

Firstly, this study adopted a cross-sectional research design, which only reveals the correlation between variables without delving into the causal mechanisms and their dynamic processes. Moreover, while the measurement tools used are highly reliable and valid, self-reported questionnaires may be subject to social desirability bias and emotional fluctuations, particularly in the subjective measurement of life satisfaction, which lacks objective data support. Future research should employ longitudinal studies or experimental designs to uncover the long-term causal effects of physical exercise on psychological variables, and incorporate wearable device records or psychophysiological indicators to comprehensively analyze the multi-level impact mechanisms of physical exercise.

Secondly, this study primarily focused on the relationships between physical exercise, self-efficacy, and resilience, but it did not fully explore the accumulation pathways of health capital and social capital. Given the multiple pressures faced by senior students, such as academic demands, employment challenges, and changes in life environments, future research could further analyze how these psychological capitals function in complex real-life contexts. Furthermore, a deeper exploration is required to understand the specific contributions of various psychological resources to enhancing life satisfaction.

Future research should aim to expand the sample scope, including more diverse cultural backgrounds and age groups, and conduct cross-group comparative studies. It should also design comprehensive intervention programs that combine physical activity and psychological skills training to better assist students in coping with the pressures of the graduation transition. At the same time, incorporating macro-level variables such as socioeconomic environment and policy support into the analysis framework would provide a theoretical basis for developing more practical mental health intervention strategies.

## 6 Conclusion

Physical exercise directly predicts self-efficacy and resilience but does not directly forecast life satisfaction. Self-efficacy does not serve as a mediator between physical exercise and life satisfaction. However, the impact of physical exercise on life satisfaction among senior students can be achieved through the mediating effect of resilience, as well as the chain mediating effect of self-efficacy and resilience. Universities should combine physical exercise with psychological skills training to help students enhance their psychological capital, alleviate the pressure of the graduation transition, and improve life satisfaction.

## Data Availability

The raw data supporting the conclusions of this article will be made available by the authors, without undue reservation.
